# Saharan Dust and Associations between Particulate Matter and Daily Mortality in Rome, Italy

**DOI:** 10.12989/ehp.1003026

**Published:** 2011-06-17

**Authors:** Sandra Mallone, Massimo Stafoggia, Annunziata Faustini, Gian Paolo Gobbi, Achille Marconi, Francesco Forastiere

**Affiliations:** 1 Institute for Cancer Prevention, Florence, Italy; 2 Department of Epidemiology, Lazio Regional Health Service, Rome, Italy; 3 Institute of Atmospheric Sciences and Climate, National Research Council, Rome, Italy; 4 Department of Environment and Primary Prevention, National Institute of Health, Rome, Italy

**Keywords:** air pollution, epidemiology, mortality, particulate matter, Saharan dust

## Abstract

Background: Outbreaks of Saharan-Sahel dust over Euro-Mediterranean areas frequently induce exceedances of the Europen Union's 24-hr standard of 50 μg/m^3^ for particulate matter (PM) with aerodynamic diameter ≤ than 10 μm (PM_10_).

Objectives: We evaluated the effect of Saharan dust on the association between different PM fractions and daily mortality in Rome, Italy.

Methods: In a study of 80,423 adult residents who died in Rome between 2001 and 2004, we performed a time-series analysis to explore the effects of PM_2.5_, PM_2.5–10_, and PM_10_ on natural, cardiac, cerebrovascular, and respiratory mortality. We defined Saharan dust days by combining light detection and ranging (LIDAR) observations and analyses from operational models. We tested a Saharan dust–PM interaction term to evaluate the hypothesis that the effects of PM, especially coarse PM (PM_2.5–10_), on mortality would be enhanced on dust days.

Results: Interquartile range increases in PM_2.5–10_ (10.8 μg/m^3^) and PM_10_ (19.8 μg/m^3^) were associated with increased mortality due to natural, cardiac, cerebrovascular, and respiratory causes, with estimated effects ranging from 2.64% to 12.65% [95% confidence interval (CI), 1.18–25.42%] for the association between PM_2.5–10_ and respiratory mortality (0- to 5-day lag). Associations of PM_2.5–10_ with cardiac mortality were stronger on Saharan dust days (9.73%; 95% CI, 4.25–15.49%) than on dust-free days (0.86%; 95% CI, –2.47% to 4.31%; *p* = 0.005). Saharan dust days also modified associations between PM_10_ and cardiac mortality (9.55% increase; 95% CI, 3.81–15.61%; vs. dust-free days: 2.09%; 95% CI, –0.76% to 5.02%; *p* = 0.02).

Conclusions: We found evidence of effects of PM_2.5–10_ and PM_10_ on natural and cause-specific mortality, with stronger estimated effects on cardiac mortality during Saharan dust outbreaks. Toxicological and biological effects of particles from desert sources need to be further investigated and taken into account in air quality standards.

Desert dust, blown by winds thousands of kilometers away from its source, represents one of the main natural contributions to atmospheric particulate matter (PM). Frequent events of dust advection from Sahara-Sahel deserts affect the Euro-Mediterranean region (Dulac et al. 1992; Gerasopoulos et al. 2006; Moulin et al. 1998). Events of dust advection have been shown to frequently induce exceedances of the World Health Organization (WHO) short-term guideline for PM with an aerodynamic diameter ≤ 10 μm (PM_10_) of 50 μg/m^3^ for a 24-hr mean, mainly in Southern Europe and with the longest duration in summer (Goudie and Middleton 2001; Rodriguez et al. 2001). The 2008 Air Quality European Union (EU) Directive (European Commission 2008) permits a maximum of 35 days exceeding the 50-μg/m^3^ 24-hr level per year, but exceedances may be excluded from the total number of days per year if they are attributable to natural sources, such as the “atmospheric re-suspension or transport of natural particles from dry regions” (European Commission 2008).

To date, the impact of dust events from deserts on health has not been extensively explored, and findings from the available studies are inconsistent. Middleton et al. (2008) found an increase in hospital admissions during dust days in the city of Nicosia (Cyprus). Studies conducted in Taipei and Seoul, Korea (mineral dust originating from the Gobi desert), have suggested an increase in total mortality related to dust episodes (Chen et al. 2004; Kwon et al. 2002). In contrast, a study conducted in the region of Greater Vancouver (British Columbia, Canada) showed no association between the Gobi dust event in 1998 and hospitalizations (Bennett et al. 2006). Schwartz et al. (1999) did not find an increase in mortality during dust days compared with control days in Spokane (WA, USA), despite the great increase in 24-hr mean PM_10_ concentration measured on dust days (263 μg/m^3^) compared with control days (42 μg/m^3^). Findings from a recent study by Perez et al. (2008) conducted in the city of Barcelona, Spain, have stimulated a debate concerning the toxicity of particles from desert sources (Sandström and Forsberg 2008; Zauli Sajani et al. 2010). Perez et al. (2008) evaluated the relationship between PM_2.5_ and PM_2.5–10_ and mortality in dust and no-dust days and found an association between coarse particles (PM_2.5–10_) and daily mortality only during Saharan dust days that they concluded might be related to biogenic agents and chemical factors carried by particles in desert dust.

We aimed to evaluate the associations between different particle fractions and daily mortality and the effect of dust advection episodes on these associations in Rome. We first estimated the effects of PM_2.5_, PM_2.5–10_, and PM_10_ on natural, cardiac, cerebrovascular, and respiratory daily mortality in Rome between 2001 and 2004. We then evaluated a dust episodes–PM interaction term to test the hypothesis that the estimated effects of PM on mortality, especially PM_2.5–10_ and PM_10_, would be higher on Saharan-dust–affected days than on other days.

## Materials and Methods

*Setting and study subjects*. The city of Rome (41°53´33.5˝N, 12°29´31˝E) is a settlement of 2.7 million inhabitants, located 23.9 km inland from the Tyrrhenian Sea, with an altitude between 13 and 140 m above sea level. Rome is affected by dust from Africa on 25–30% of days. Most of these episodes occur from May to October, and dust reaches the ground on a large proportion of the days (Gobbi et al. 2006, 2007; Matassoni et al. 2009). Rome has relatively high levels of PM_10_, and an association between PM_10_ and daily mortality has been already shown (Forastiere et al. 2008).

The study subjects were 80,423 residents ≥ 35 years of age who died within the city from natural causes during 14 February 2001 to 31 December 2004. Data were obtained from the Regional Register of Causes of Deaths, in which death causes are coded according to the *International Classification of Diseases*, version 9 (ICD-9) (WHO 1975). All natural causes (ICD-9 codes 1–799), circulatory system causes (ICD-9 codes 390–459), and respiratory system causes (ICD-9 codes 460–519) were analyzed. Within the circulatory system group, deaths due to cardiac causes (ICD-9 390–429) and cerebrovascular causes (ICD-9 430–438) specifically were also considered.

We obtained data on annual influenza epidemics (the annual 3-week period of maximum incidence of flulike illness) based on estimates of weekly influenza incidence as reported by the Italian National Health Surveillance System.

*Pollutant and meteorological data.* Data on PM_10_ were provided by the Lazio Environmental Protection Agency (EPA) and collected according to standard procedures (Katsouyanni et al. 1996). Data on PM_2.5_ were not regularly monitored by the regional EPA during the study period, so we used a monitoring station 2 km east of the city center on the grounds of the Italian National Institute of Health (NIH). The same monitoring station provided data for PM_10_ (Cattani et al. 2010). Gravimetric measurement methods were used to determine the PM_10_ mass fraction of suspended PM (European Commission 1999); a provisional method was applied for PM_2.5_ that follows the standards set in 2005 (Comité Européen de Normalisation 2005).

Hourly PM_10_, nitrogen dioxide (NO_2_), and ozone (O_3_) data from the Lazio EPA were measured at three fixed stations in residential areas. PM_10_ was measured using the beta array attenuation, a method that operates by drawing air through a continuous glass or tape; beta particles are then passed through the particles deposited on the tape, and the attenuation of these particles is measured in a sensor. The attenuation is converted to an estimate of mass based on the absorption coefficient. The 24-hr mean daily value was considered for PM_10_ and NO_2_, whereas the daily maximum 8-hr running mean during the warm season (April–September) was chosen for O_3_. To account for missing data for a specific monitor per pollutant per day, we used information on the same pollutant and monitor on other days of the same year, plus measurements of the same pollutant and day on the other available monitors, as described elsewhere (Biggeri et al. 2004).

The 24-hr daily mean value was considered also for PM_10_ and PM_2.5_ measured at the NIH monitoring station. In this case, to impute missing PM_10_ data, we used daily concentrations from the regional monitoring network, whereas missing data for PM_2.5_ were imputed using season-specific PM_2.5_–PM_10_ regression values from the PM_10_ series measured at the NIH station (Belleudi et al. 2010). A total of 601 PM_10_ and 177 PM_2.5_ data points were estimated. Coarse PM concentrations (PM_2.5–10_) were thus obtained by subtracting 24-hr mean value of PM_2.5_ from the corresponding PM_10_ value collected at the same NIH monitoring station.

In summary, we evaluated the following three PM exposure variables: PM_10_ (from the regional monitoring network), PM_2.5_ (from the NIH monitor), and PM_2.5–10_ (from the NIH monitor). PM_10_ from the NIH monitoring station was used only to derive the coarse (PM_2.5–10_) fraction, because it displayed a close agreement with the regional series (PM_10-NIH_: mean ± SD, 40.0 ± 17.7 μg/m^3^; PM_10-EPA_: 39.9 ± 16.5 μg/m^3^; Pearson correlation between the two PM_10_ series, 0.85).

We obtained daily information on temperature, dew-point temperature, relative humidity, and sea-level barometric pressure for 2001–2004 from the Italian Air Force Meteorological Service. Apparent temperature was calculated on the basis of air temperature and dew-point temperature according to the following formula:

Apparent temperature =  –2.653 + 0.994 temperature  + 0.0153(dew point temperature squared).

*Saharan dust days.* Starting on 14 February 2001, a polarization LIDAR was operated at the Institute of Atmospheric Sciences and Climate (ISAC) laboratories, located at the southern outskirts of Rome (Rome Tor Vergata, 41°8´40˝N, 12°6´47˝E). LIDAR (or laser radar) is a technology that analyzes the atmospheric return of laser pulses to generate a time–altitude structural picture of a distant target. Because of its high sensitivity to atmospheric particles and gases, the LIDAR technique is extensively used for atmospheric environment and meteorology research. Polarization LIDARs can distinguish nonspherical particles such as Saharan dust from commonly spherical (liquid) PM. A detailed description of the ISAC-Rome LIDAR system and data analysis has been given elsewhere (Gobbi et al. 2004, 2007). A dust index (DI; 0 or 1) was calculated on the basis of LIDAR observations, validated, and complemented by Web-accessible analysis products of operational models, such as the dust regional atmospheric model (DREAM) of the Barcelona Supercomputing Centre (BSC 2010) and the U.S. Navy Aerosol Analysis and Prediction System (Naval Research Laboratories 2010; see, e.g., Gobbi et al. 2006).

Because Saharan dust events in Rome were found to bring an optically meaningful layer affecting the region up to 6 km above the ground (Gobbi et al. 2004), our definition of dust-affected days was related to a DI of 1 and a PM_10_:NO_2_ ratio > 0.6. PM_10_:NO_2_ ratios ≤ 0.6 are typical of many Italian cities where traffic is the main source of air pollution (Berti et al. 2009). Therefore, this definition restricted our analyses to days with an objective dust layer over the city (at any altitude) and a higher ground-level PM_10_ concentration than would be expected from traffic-related air pollution only.

*Statistical analysis.* For the statistical analysis, we used a time-stratified case-crossover design (Levy et al. 2001; Maclure 1991) in which the exposure of the index day of the health event is compared with the exposure during one or more control periods. We followed a time-stratified approach by dividing the study period into monthly strata and selecting control days to be all days falling on the same days of the week within the same stratum as the index day. We fitted a basic Poisson regression model to the daily mortality data that included potential confounders: weather, indicator variables reflecting the changes in the size of the population at risk, influenza epidemics, and time trends and seasonality. Two different penalized splines were built to adjust for the potential confounding of low and high temperatures: for the cold period at lag 1–6 of air temperature (days on which air temperature is below the city-specific median value, calculated on the all-year time series) and for the warm period at lag 0–1 of apparent temperature (days on which apparent temperature is above the all-year median). In addition, the nonlinear effect of barometric pressure was controlled for with a penalized spline (lag 0). We also controlled for population decrease during holidays and in summer months, and for influenza epidemics, with categorical variables. Finally, we included a three-way interaction term between day of the week, month, and year to control for both seasonality and time trends. This choice was motivated by the need to replicate the adjustment made by the case-crossover design with the “time-stratified” approach for the selection of control days (Lu and Zeger 2007). To take into account possible overdispersion of daily counts of deaths, we used quasi-likelihood estimation. We used separate models to estimate effects of PM_2.5_, PM_2.5–10_, and PM_10_ on total and cause-specific daily mortality. Exposures were averaged over the following *a priori* lag periods on the basis of previous work (Stafoggia et al. 2009): lag 0–2 for natural, cardiac, and circulatory system mortality, lag 0 for cerebrovascular mortality, and lag 0–5 for respiratory mortality. We then evaluated whether the PM–mortality association was modified by Saharan dust by including an interaction term between the indicator variable of Saharan dust days and PM_2.5_, PM_10_, and PM_2.5–10_, respectively. Estimated effects are reported as percentage increases in risk of death (IR%) and 95% confidence intervals (CIs) associated with an interquartile range (IQR; 25th to 75th percentile) increase in each pollutant.

We conducted a number of additional analyses. First, we considered the potential confounding effect of O_3_ during the warm season (April to September). Second, we evaluated whether associations between PM and cause-specific mortality were different according to the duration of Saharan dust episodes. Third, we performed two sensitivity analyses to check the robustness of the main results to high-temperature adjustment: *a*) by adding to the base model a spline term for lag 2–6 apparent temperature in order to consider a longer latency of the effect of summer apparent temperature on mortality; and *b*) by restricting the analysis to days below the 95th percentile of the apparent temperature distribution in order to check the robustness of the main results to extreme summer days. Finally, we investigated the association between PM_10_ and cause-specific mortality using the particle concentrations measured at the NIH monitoring station as alternative to the regional EPA measurements.

Daily mortality, pollutant, and meteorological data were converted into a STATA 10 data set (StataCorp LP, College Station, TX, USA). All statistical analyses were run in R software (version 2.6.2; R Foundation for Statistical Computing, Vienna, Austria) using the generalized additive model procedure. We considered statistically significant all results with α < 0.05 (95% CI excluding 1), and all interaction terms with *p* < 0.15.

## Results

*Saharan dust days, pollutants, and meteorological data.* During 2001–2004, Saharan dust affected 18.6% of days in the city of Rome (264 days). The proportion of days with a dust episode was maximal in the warmer seasons (26.1% of summer days, 22.6% of spring days) and lowest in the colder seasons (17.6% of fall days, 6.2% of winter days). The duration of each Saharan event was between 1 and 9 days (data not shown). The mean concentrations of all fractions of particles were higher during dust days than during dust-free days, but the increase was much more pronounced for PM_2.5–10_ (20.7 μg/m^3^ vs. 14.6 μg/m^3^) than for PM_2.5_ (25.6 vs. 23.4 μg/m^3^; Table 1). Correlation coefficients between PM_2.5–10_ and EPA PM_10_ were 0.54 for dust-free days and 0.82 for dust-affected days, and correlations between PM_2.5_ and PM_2.5–10_ were 0.27 and 0.18 for dust-free and dust-affected days, respectively (data not shown). The PM_10_:NO_2_ ratio increased from a mean ± SD of 0.6 ± 0.2 on dust-free days to 0.9 ± 0.4 on dust-affected days. As expected, air temperature, apparent temperature, and O_3_ also were higher during dust-affected days than during other days (Table 1).

**Table 1:** Pollutants and meteorological data during dust-free and dust-affected days: Rome, 2001–2004.

Percentile				
Environmental variable	Mean ± SD		Minimum	25th	50th	75th	IQR	Maximum	*n*
Saharan-dust–free days (1,153 days)																	
PM_2.5_ (μg/m^3^)*a*		23.4 ±	12.5		2.4		15.0		20.9		28.2		13.2		91.7		873
PM_2.5–10_ (μg/m^3^)*a*		14.6 ±	8.7		0.0		8.8		13.6		19.1		10.3		60.8		853
PM_10_ (μg/m^3^)*a*		38.4 ±	17.0		6.7		27.5		34.8		44.1		16.6		115.2		1,086
PM_10_ (μg/m^3^)*b*		37.1 ±	14.8		9.7		26.9		34.5		44.5		17.6		110.4		1,153
NO_2_ (μg/m^3^)*b*		63.1 ±	15.6		19.5		51.7		62.6		73.8		22.1		117.9		1,151
PM_10_:NO_2_*b*		0.6 ±	0.2		0.2		0.5		0.6		0.7		0.2		2.8		1,151
O_3_ (μg/m^3^)*b*		75.5 ±	38.6		7.0		43.7		76.5		102.6		58.9		199.2		1,148
Air temperature (°C)		15.4 ±	7.0		–0.4		9.9		15.1		21.1		11.3		30.8		1,153
Relative humidity (%)		76.1 ±	13.4		33.5		67.1		78.4		87.4		20.3		98.6		1,153
Barometric pressure (hPa)		1015.1 ±	6.6		992.5		1011.1		1015.4		1019.0		7.8		1036.0		1,153
Apparent temperature (°C)		15.1 ±	8.5		–2.8		8.0		14.4		22.4		14.4		33.2		1,153
Saharan-dust–affected days (264 days)																	
PM_2.5_ (μg/m^3^)*a*		25.6 ±	9.8		5.6		19.0		24.0		30.5		11.5		86.0		216
PM_2.5–10_ (μg/m^3^)*a*		20.7 ±	12.9		3.3		14.3		18.3		23.4		9.2		101.9		213
PM_10_ (μg/m^3^)*a*		47.2 ±	18.8		19.0		35.4		43.6		54.2		18.8		156.9		253
PM_10_ (μg/m^3^)*b*		52.2 ±	18.5		21.8		41.4		49.9		58.9		17.5		181.7		264
NO_2_ (μg/m^3^)*b*		60.4 ±	15.6		26.0		48.3		60.3		70.3		22.0		102.0		263
PM_10_:NO_2_*b*		0.9 ±	0.4		0.6		0.7		0.8		1.0		0.3		3.4		263
O_3_ (μg/m^3^)*b*		85.6 ±	36.9		5.9		56.9		91.7		110.7		53.8		163.6		262
Air temperature (°C)		20.2 ±	5.6		6.0		15.7		20.7		25.2		9.5		29.7		264
Relative humidity (%)		74.0 ±	14.4		31.0		63.2		76.1		86.2		23.0		97.2		264
Barometric pressure (hPa)		1014.7 ±	5.6		994.6		1011.9		1014.8		1017.4		5.5		1032.2		264
Apparent temperature (°C)		21.1 ±	7.0		3.6		15.7		21.4		27.4		11.7		33.3		264
**a**Data from the Italian NIH monitoring station. **b**Data from the regional EPA monitoring stations.																	

*Association of PM with mortality and effect modification.* Table 2 displays the absolute and daily mean number of deaths by season in dust days and other days, as well as overall. Of 80,423 deaths from nonaccidental causes among city residents ≥ 35 years of age, 42.0% were due to circulatory system diseases (30.8% due to cardiac diseases, 9.2% due to cerebrovascular diseases) and 5.7% were due to respiratory system diseases. The daily mean number of deaths for natural causes was similar on dust and nondust days when considering the year-round distribution, although substantial differences emerged by season, with higher mortality in winter and in summer during dust days than during dust-free days.

**Table 2:** Study subjects and daily mean numbers of deaths by cause of death, season, and Saharan-dust–affected and free days: Rome, 2001–2004.

Saharan-dust–free days			Saharan-dust–affected days			All days		
Cause of death/season	*n*	Daily mean	*n*	Daily mean	*n*	Daily mean
Natural (ICD-9 codes 1–799)		65,469		56.8		14,954		56.6		80,423		56.8
Winter		18,622		62.9		1,355		64.5		19,977		63.0
Spring		16,320		57.3		4,707		56.7		21,027		57.1
Summer		14,546		53.5		5,514		57.4		20,060		54.5
Fall		15,981		53.3		3,378		52.8		19,359		53.2
Cardiac (ICD-9 codes 390–429)		20,199		17.5		4,574		17.3		24,773		17.5
Winter		6,045		20.4		447		21.3		6,492		20.5
Spring		5,200		18.2		1,444		17.4		6,644		18.1
Summer		4,072		15.0		1,658		17.3		5,730		15.6
Fall		4,882		16.3		1,025		16.0		5,907		16.2
Cerebrovascular (ICD-9 codes 430–438)		6,015		5.2		1,424		5.4		7,439		5.2
Winter		1,760		5.9		131		6.2		1,891		6.0
Spring		1,404		4.9		438		5.3		1,842		5.0
Summer		1,424		5.2		548		5.7		1,972		5.4
Fall		1,427		4.8		307		4.8		1,734		4.8
Circulatory system (ICD-9 codes 390–459)		27,497		23.8		6,262		23.7		33,759		23.8
Winter		8,198		27.7		598		28.5		8,796		27.7
Spring		6,910		24.2		1,983		23.9		8,893		24.2
Summer		5,761		21.2		2,288		23.8		8,049		21.9
Fall		6,628		22.1		1,393		21.8		8,021		22.0
Respiratory system (ICD-9 codes 460–519)		3,736		3.2		838		3.2		4,574		3.2
Winter		1,203		4.1		84		4.0		1,287		4.1
Spring		982		3.4		269		3.2		1,251		3.4
Summer		732		2.7		317		3.3		1,049		2.9
Fall		819		2.7		168		2.6		987		2.7


Table 3 reports the estimated effects of different PM fractions on mortality outcomes and effect modification by the indicator of dust days. A 12.8 μg/m^3^ rise in PM_2.5_ (IQR) was associated with a 1.24% (95% CI, –0.65% to 3.17%) increase in the overall mortality and a 1.23% (95% CI, –1.42% to 3.95%) increase in mortality caused by diseases of the circulatory system. All other effect estimates were positive, except for cerebrovascular disease, but were not statistically significant (*p* > 0.05). We observed no modification of the estimated effect of fine particles (PM_2.5_) on mortality (either total or cause specific) during dust days, with *p*-values of the interaction terms always > 0.30.

**Table 3:** IR% and 95% CI associated with IQR increases in different PM fractions by cause of death and the presence or absence of Saharan dust.

PM_2.5_ (IQR = 12.8 μg/m^3^)			PM_2.5–10_ (IQR = 10.8 μg/m^3^)			PM_10_ (IQR = 19.8 μg/m^3^)		
Cause of death	IR% (95% CI)	*p*-Value	IR% (95% CI)	*p*-Value	IR% (95% CI)	*p*-Value
Natural causes (lag 0–2) (ICD-9 codes 1–799)		1.24 (–0.65 to 3.17)				2.96 (1.23 to 4.72)				3.04 (1.53 to 4.56)		
Dust free		0.91 (–1.09 to 2.94)		—		3.33 (1.29 to 5.40)		—		3.01 (1.29 to 4.75)		—
Dust affected		3.22 (–1.13 to 7.75)		0.316		2.07 (–1.07 to 5.31)		0.502		3.20 (–0.04 to 6.55)		0.914
Cardiac diseases (lag 0–2) (ICD-9 codes 390–429)		1.38 (–1.68 to 4.55)				3.72 (0.78 to 6.73)				4.04 (1.49 to 6.65)		
Dust free		0.97 (–2.24 to 4.29)		—		0.86 (–2.47 to 4.31)		—		2.09 (–0.76 to 5.02)		—
Dust affected		1.37 (–5.92 to 9.21)		0.920		9.73 (4.25 to 15.49)		0.005		9.55 (3.81 to 15.61)		0.019
Cerebrovascular diseases (lag 0) (ICD-9 codes 430–438)		–0.32 (–4.26 to 3.78)				5.41 (1.74 to 9.22)				2.64 (–1.26 to 6.68)		
Dust free		–0.07 (–4.29 to 4.33)		—		4.58 (–0.18 to 9.58)		—		3.24 (–1.78 to 8.51)		—
Dust affected		–3.22 (–11.72 to 6.10)		0.514		7.03 (1.22 to 13.18)		0.526		1.71 (–5.05 to 8.95)		0.720
Diseases of the circulatory system (lag 0–2) (ICD-9 codes 390–459)		1.23 (–1.42 to 3.95)				4.06 (1.50 to 6.69)				2.99 (0.82 to 5.19)		
Dust free		1.19 (–1.60 to 4.05)		—		2.21 (–0.74 to 5.25)		—		1.82 (–0.61 to 4.32)		—
Dust affected		–0.91 (–7.04 to 5.62)		0.531		7.93 (3.20 to 12.88)		0.039		5.91 (1.02 to 11.03)		0.134
Diseases of the respiratory system (lag 0–5) (ICD-9 codes 460–519)		0.25 (–9.90 to 11.54)				12.65 (1.18 to 25.42)				4.97 (–2.18 to 12.63)		
Dust free		–2.17 (–12.72 to 9.66)		—		8.67 (–4.14 to 23.19)		—		5.00 (–2.80 to 13.43)		—
Dust affected		8.38 (–12.66 to 34.49)		0.368		19.43 (0.34 to 42.15)		0.349		2.66 (–12.78 to 20.83)		0.798
The *p*-value is for the interaction between PM and the Saharan dust indicator. PM_2.5_ and PM_2.5–10_ data are from the Italian NIH monitoring station, and PM_10_ data are from regional EPA monitoring stations. Results from multivariate Poisson regression models are adjusted for time trend, seasonality, day of the week, summer population decrease, holidays, influenza epidemics, high apparent temperatures (penalized spline, lag 0–1), low air temperatures (penalized spline, lag 1–6), and barometric pressure (penalized spline, lag 0).												

The estimated effects of a 10.8-μg/m^3^ rise in PM_2.5–10_ ranged from a 2.96% (95% CI, 1.23–4.72%) increase for natural mortality to a 12.65% (95% CI, 1.18–25.42%) increase for respiratory mortality. Associations between PM_2.5–10_ and cerebrovascular (5.41%; 95% CI, 1.74–9.22%), and circulatory (4.06%; 95% CI, 1.50–6.69%) mortality were also positive and statistically significant. The estimated effects of PM_2.5–10_ on circulatory mortality, and on cardiac mortality specifically, were much stronger during dust-affected days (9.73%; 95% CI, 4.25–15.49%) than during dust-free days (0.86%; 95% CI, –2.47% to 4.31%; *p* = 0.005). Respiratory mortality also was increased from 8.67% (95% CI, –4.14% to 23.19%) on dust-free days to 19.43% (95% CI, 0.34–42.15%) on dust-affected days, providing a suggestion of effect modification, even though the interaction was not significant (*p* = 0.35).

A 19.8-μg/m^3^ rise in PM_10_ was associated with a 3.04% (95% CI, 1.53–4.56%) increase in total mortality and a 4.04% (95% CI, 1.49–6.65%) increase in cardiac mortality. Saharan dust modified the estimated effect of PM_10_ on cardiac mortality (9.55%; 95% CI, 3.81–15.61%; vs. dust-free days: 2.09%; 95% CI, –0.76% to 5.02%; *p* = 0.019) and circulatory mortality (5.91%; 95% CI, 1.02–11.03%; vs. 1.82%; 95% CI, –0.61% to 4.32%; *p* = 0.134).

The main results of the association between particles and mortality, and the effect modification by Saharan dust days, were robust to O_3_ adjustment [see Supplemental Material, Table 1 (http://dx.doi.org/10.1289/ehp.1003026)]. Similarly, the exclusion of extremely warm days (> 29°C apparent temperature) from analyses did not affect estimated effects of particles on mortality overall or effect modification by the Saharan dust indicator. The duration of Saharan dust episodes also did not affect the association between different PM fractions and cause-specific mortality (see Supplemental Material, Table 2). Finally, adjusting for high apparent temperatures using two spline terms instead of one, and the use of PM_10_ from the Italian NIH as an alternative to the regional EPA measurements, gave results very similar to those obtained in the main analysis (data not shown).

## Discussion

We found strong and statistically significant associations between IQR increases in PM_2.5–10_ and daily mortality in Rome, particularly for mortality due to circulatory and respiratory diseases. In comparison, associations between mortality and IQR increases in PM_2.5_ were weaker. We observed a clear effect modification for cardiac mortality, with stronger estimated effects of PM_2.5–10_ during Saharan dust days than during dust-free days.

The health effects of coarse particles (PM_2.5–10_) are still controversial. These particles were generally believed to be less harmful than finer particles. However, the review of Brunekreef and Forsberg (2005) pointed out the need for further study of the health effects of PM_2.5–10_ because most of the available studies were not able to isolate an independent effect for coarse particles from that of fine PM. Coarse particles have been shown to have the same toxicological capacity as PM_2.5_ on a mass basis in European studies (Gerlofs-Nijland et al. 2007; Sandström et al. 2005). However, Monn and Becker (1999) evaluated water-soluble components of ambient PM_2.5_ and PM_10_ (both indoor and outdoor) and showed that cytokine production by human monocytes [interleukin (IL)-6 and IL-8] *in vitro* increased in response to eluates of PM_10_ only. Other experimental studies have shown that coarse particle extracts induced greater cytotoxicity and production of proinflammatory cytokines (tumor necrosis factor-α, monocyte chemotactic protein-1, and IL-6) than did fine particle extracts (Becker et al. 2003; Soukup and Becker 2001). Becker et al. (2003) hypothesized that these findings could be due to an inhibitory effect of coarse PM on the antimicrobial activity of alveolar macrophages, or an effect on cytokine or growth factor production resulting in inflammation and tissue remodeling.

Our results suggest a stronger effect for PM_2.5–10_ than for PM_2.5_ or total PM_10_ on circulatory and respiratory mortality, indicating that coarse particles exposures might pose great health risks. The findings are similar to those reported by Villeneuve et al. (2003) for cardiovascular mortality in Vancouver, Canada. Ostro et al. (2003) reported a significant association between PM_2.5–10_ (but not PM_2.5_) and cardiac mortality in Coachella Valley, California, where coarse PM exposure levels were high (daily mean concentration, 25.8 μg/m^3^). Malig and Ostro (2009) found evidence of an association between acute exposure to coarse particles and both all-cause and cardiovascular mortality in a multicity study in California. Associations between PM_2.5–10_ and respiratory mortality have been reported in the analysis done by Zanobetti and Schwartz (2009) on 112 U.S. counties, and in a study from Mexico City (Castillejos et al. 2000) where the estimated effect of coarse particles was greater than that of fine particles. However, Schwartz et al. (1996) and Kan et al. (2007) did not find significant associations between PM_2.5–10_ and cardiovascular and ischemic mortality, respectively.

The analysis of effect modification of desert dust on the PM–mortality association clearly showed stronger estimated effects of PM_2.5–10_ and PM_10_ on cardiac and circulatory mortality during Saharan dust days events than during other days. The main question that remains to be answered is whether this reflects an effect of the Saharan dust per se, or effects of specific components of Saharan dust, including biological material or anthropogenic pollutants collected during the long-distance transport, or a synergistic effect of Saharan dust in combination with other pollutants.

Few data are available on PM composition during Saharan dust days in Rome, and these are usually related to tracing indicators for different natural sources. A quantitative estimate of five main fractions of atmospheric PM (crustal, sea-salt, primary anthropogenic, secondary inorganic, and organic matter) was carried out in Rome during six episodes of dust between October 2004 and July 2005 (Perrino et al. 2009). The results showed that desert dust and sea aerosol were the major contributors to coarse particles in Rome; wildfires were sporadic contributors; and PM emission from Etna, the main Mediterranean volcano sited in Sicily, was limited in time and had little influence on PM levels in central Italy. The nature of a Saharan event was confirmed by backward trajectories calculated using the National Oceanic and Atmospheric Administration HYSPLIT (Hybrid Single Particle Lagrangian Integrated Trajectory) model (Air Resources Laboratory 2010) and DREAM, which predicts the atmospheric life cycle of eroded desert dust (BSC 2010). Saharan dust episodes modified PM fractions, increasing the proportion of coarse particles relatively to fine particles. As in the work from Perez et al. (2008) in Barcelona, the contribution from mineral dust and other crustal materials was indicated mainly by the presence of silicon, manganese, and titanium (Perrino et al. 2007).

We applied back-trajectories to nondust and dust days in our study to confirm the Saharan origin of the PM on dust days. We computed 10-day back-trajectories ending in the Rome planetary boundary layer for each day (i.e., 1,460 runs) of the 4-year period by means of the HYSPLIT code (Air Resources Laboratory 2010). The results of this analysis clearly indicate the shift in the origin of air masses reaching Rome from a Mediterranean sea origin on nondust days to a northern Africa origin on dust days [see Supplemental Material, Figure 1 (http://dx.doi.org/10.1289/ehp.1003026)]. The most important sources of mineral dust reaching Rome were the desert regions of southern Tunisia (Grand Erg Oriental) and their borders with the Algerian and Libyan deserts.

PM composition and its biological properties may be different during dust days. Two biological studies on Saharan desert dust transported to Haifa, Israel, and Erdemli, Turkey, showed that microorganisms are able to survive long-range transport and to represent a risk for human health in the arrival areas (Griffin et al. 2007; Schlesinger et al. 2006). Two studies conducted in four European cities (Steerenberg et al. 2006) and in the United States (Gilmour et al. 2007) have suggested that exposure to crustal-derived PM is associated with inflammatory response and acute toxicity. Also, anthropogenic pollutants that may adhere to crustal-derived PM as it passes through areas may play a role in exacerbating inflammatory and immunogenic responses.

We do not have complete data on metals, microorganisms, and endotoxins or on the copresence of other pollutants (e.g., volatile organic compounds) in PM during Saharan dust days, so we cannot determine specific components that could account for stronger estimated effects on dust days or confirm the natural or anthropogenic origin of PM. Inconsistencies in the existing evidence suggest that characteristics associated with the short-term effects of desert dust should be further studied, with special attention to their composition, biological properties, and anthropogenic components that may be associated with the dust particles.

Because of the small number of dust days, we were not able to evaluate other individual potential effect modifiers (e.g., sex, age). Because the concentration of coarse particles is indirectly determined (by subtracting the measured concentration of PM_2.5_ from that of PM_10_), it may be affected by errors in the measurement of both PM fractions. This might have resulted in a reduction of chance in detecting an existing association. On the other hand, adjustment for O_3_ levels and the use of alternative models to adjust for high summer temperatures did not have a substantial consequence on estimated effects.

## Conclusions

Our results add to existing evidence indicating that short-term changes in PM_2.5–10_ and PM_10_ increase the risk of cerebrovascular, circulatory, and respiratory mortality. We estimated the strongest associations for coarse particles (PM_2.5–10_). We also observed evidence of stronger effects of PM_2.5–10_ and PM_10_ on cardiac and circulatory mortality during Saharan dust episodes, which highlights the need for additional research on the toxicological and inflammatory effects of particles derived from desert sources. Air quality standards should consider the potential health effects of dust from natural sources.

## Supplemental Material

(312 KB) PDF
