# Supplementary material for: Saharan Dust and Associations between Particulate Matter and Daily Mortality in Rome, Italy
Source: Environ Health Perspect. 2011 Jun 17;119(10):1409–14. doi: 10.12989/ehp.1003026 (PMC3230430; doi:10.12989/ehp.1003026)
Supplement: (312 KB) PDF [file ehp.1003026.s001.508.pdf]

Supplemental Material

Saharan Dust and Associations between Particulate Matter and Daily Mortality in Rome, Italy

Sandra Mallone, Massimo Stafoggia, Annunziata Faustini, Gian Paolo Gobbi, Achille Marconi and Francesco Forastiere

Corresponding author:

Massimo Stafoggia

Department of Epidemiology, Lazio Regional Health Service, Via Santa Costanza 53, 00198, Rome, Italy

Phone: +390683060474

Fax: +390683060374

Email: stafoggia@asplazio.it

Table of Contents

Tables

Figures

Supplemental Material, Table 1. Percentage change in risk of death (IR %) and 95% confidence interval (95% CI) associated with IQR increases in different PM fractions by cause of death and the presence or absence of Saharan dust: results of the adjustment for ozone (April to September)

Cause of death	$PM_{2.5}$ (IQR = 12.8 µg/m^3)			$PM_{2.5\text{-}10}$ (IQR = 10.8 µg/m^3)			PM_{10} (IQR = 19.8 µg/m^3)		
	IR%	95% CI	p-value	IR%	95% CI	p-value	IR%	95% CI	p-value
Natural causes (lag 0-2) (ICD9: 1-799)									
Saharan Dust Days									
Dust-free	0.78	(-1.20 , 2.81)	-	3.43	(1.41 , 5.50)	-	3.00	(1.30 , 4.73)	-
Dust affected	2.49	(-1.84 , 7.02)	0.457	1.81	(-1.34 , 5.05)	0.387	3.18	(-0.07 , 6.53)	0.923
Cardiac diseases (lag 0-2) (ICD9: 390-429)									
Saharan Dust Days									
Dust-free	0.89	(-2.32 , 4.20)	-	0.97	(-2.37 , 4.42)	-	2.11	(-0.74 , 5.05)	-
Dust affected	0.51	(-6.75 , 8.33)	0.923	9.35	(3.84 , 15.15)	0.009	9.41	(3.64 , 15.51)	0.022
Cerebrovascular diseases (lag 0) (ICD9: 430-438)									
Saharan Dust Days									
Dust-free	-0.29	(-4.51 , 4.12)	-	4.54	(-0.22 , 9.54)	-	3.07	(-1.94 , 8.34)	-
Dust affected	-4.07	(-12.55 , 5.23)	0.432	6.97	(1.15 , 13.13)	0.531	1.56	(-5.22 , 8.81)	0.722
Diseases of the circulatory system (lag 0-2) (ICD9: 390-459)									
Saharan Dust Days									
Dust-free	1.08	(-1.69 , 3.93)	-	2.33	(-0.62 , 5.36)	-	1.91	(-0.53 , 4.41)	-
Dust affected	-1.99	(-8.07 , 4.49)	0.357	7.45	(2.70 , 12.42)	0.066	5.55	(0.65 , 10.70)	0.182
Diseases of the respiratory system (lag 0-5) (ICD9: 460-519)									
Saharan Dust Days									
Dust-free	-2.24	(-12.80 , 9.60)	-	8.18	(-4.61 , 22.68)	-	5.10	(-2.73 , 13.55)	-
Dust affected	9.71	(-11.77 , 36.41)	0.316	20.95	(1.46 , 44.18)	0.273	3.47	(-12.16 , 21.90)	0.861

IR - Increase in Risk; CI - Confidence Interval; IQR - Interquartile Range; $PM_{2.5}$ - Particulate matter with diameter < 2.5 microns; $PM_{2.5\text{-}10}$ - Particulate matter with diameter between 2.5 and 10 microns; PM_{10} - Particulate matter with diameter < 10 microns; ICD9 - International Classification of Diseases, 9th revision

p-value: p-value of the interaction between PM and the Saharan dust indicator.

$PM_{2.5}$ and $PM_{2.5\text{-}10}$ data from NIH monitoring station; PM_{10} data from regional EPA monitoring stations**.**

Results from multivariate Poisson regression models adjusted for time trend, seasonality, day of the week, summer population decrease, holidays, apparent temperature (penalized spline, lag 0-1), barometric pressure (penalized spline, lag 0).

Supplemental Material, Table 2. Percentage increase in risk of death (IR %) and 95% confidence interval (95% CI) per an interquartile range (IQR) change in $PM_{2.5-10}$ and PM_{10}, by cause of death and Saharan dust. Results of the sensitivity analysis (exclusion of days with apparent temperature above 29°C) and the additional analysis (classification of dust days by duration of dust episodes)

	Exclusion of day with apparent temperature >29°C					Classification of dust days by duration of dust episodes				
	Dust	IR%	95% CI		p-value	Dust	IR%	95% CI		p-value
**$PM_{2.5-10}$, IQR=10.8 μg/m^3**										
Natural causes (lag 0-2)		**2.92**	**1.19**	**4.69**	-		**2.96**	**1.23**	**4.72**	
	Dust-free	3.34	1.31	5.41	-	*Dust-free*	3.22	1.18	5.29	-
	Dust affected	1.94	-1.23	5.22	0.457	≤ 3 days	2.36	-1.67	6.55	0.708
						> 3 days	1.71	-3.13	6.78	0.579
Cardiac diseases (lag 0-2)		**3.14**	**0.20**	**6.15**	-		**3.72**	**0.78**	**6.73**	
	Dust-free	0.67	-2.65	4.11	-	*Dust-free*	0.84	-2.49	4.28	-
	Dust affected	8.99	3.46	14.81	0.009	≤ 3 days	7.80	0.97	15.09	0.068
						> 3 days	12.25	3.55	21.68	0.015
Cerebrovascular diseases (lag 0)		**5.76**	**1.93**	**9.74**	-		**5.41**	**1.74**	**9.22**	
	Dust-free	4.72	-0.26	9.95	-	*Dust-free*	4.57	-0.20	9.58	-
	Dust affected	7.20	1.16	13.60	0.536	≤ 3 days	7.18	-0.08	14.96	0.564
						> 3 days	6.79	-2.42	16.87	0.682
Diseases of the circulatory system (lag 0-2)		**3.82**	**1.26**	**6.45**	-		**4.06**	**1.50**	**6.69**	
	Dust-free	2.21	-0.72	5.24	-	*Dust-free*	2.20	-0.75	5.23	-
	Dust affected	7.38	2.61	12.37	0.062	≤ 3 days	7.11	1.14	13.44	0.143
						> 3 days	8.78	1.37	16.73	0.103
Diseases of the respiratory system (lag 0-5)		**11.90**	**0.13**	**25.06**	-		**12.65**	**1.18**	**25.42**	
	Dust-free	8.27	-4.93	23.29	-	*Dust-free*	8.63	-4.20	23.18	-
	Dust affected	19.66	0.04	43.14	0.335	≤ 3 days	18.92	-2.76	45.42	0.421
						> 3 days	19.12	-15.91	68.73	0.619

PM_{10}, IQR=19.8 µg/m^3

Natural causes (lag 0-2)		**3.03**	**1.54**	**4.55**	-		**3.00**	**1.51**	**4.51**	
	Dust-free	2.88	1.19	4.59	-	*Dust-free*	2.84	1.14	4.56	-
	Dust affected	2.76	-0.62	6.25	0.950	≤ 3 days	3.89	-0.33	8.28	0.648
						> 3 days	3.06	-1.87	8.25	0.932
Cardiac diseases (lag 0-2)		**3.36**	**0.85**	**5.93**	-		**3.62**	**1.11**	**6.18**	
	Dust-free	1.82	-1.00	4.71	-	*Dust-free*	1.63	-1.18	4.51	-
	Dust affected	8.84	2.85	15.18	0.032	≤ 3 days	9.16	1.91	16.93	0.055
						> 3 days	11.42	2.38	21.26	0.040
Cerebrovascular diseases (lag 0)		**3.10**	**-0.86**	**7.21**	-		**3.01**	**-0.84**	**7.02**	
	Dust-free	3.47	-1.55	8.76	-	*Dust-free*	3.47	-1.49	8.68	-
	Dust affected	0.35	-6.74	7.98	0.480	≤ 3 days	0.92	-7.12	9.67	0.605
						> 3 days	3.30	-8.27	16.33	0.979
Diseases of the circulatory system (lag 0-2)		**2.85**	**0.73**	**5.02**	-		**2.88**	**0.75**	**5.06**	
	Dust-free	1.94	-0.46	4.39	-	*Dust-free*	1.76	-0.65	4.23	-
	Dust affected	4.92	-0.13	10.22	0.285	≤ 3 days	5.31	-0.91	11.91	0.297
						> 3 days	7.92	0.26	16.18	0.130
Diseases of the respiratory system (lag 0-5)		**3.42**	**-3.70**	**11.06**	-		**4.57**	**-2.46**	**12.09**	
	Dust-free	3.64	-4.12	12.03	-	*Dust-free*	4.57	-3.15	12.91	-
	Dust affected	2.67	-14.19	22.86	0.922	≤ 3 days	-2.15	-20.25	20.07	0.540
						> 3 days	10.94	-14.42	43.81	0.663

IR - Increase in Risk; CI - Confidence Interval; $PM_{2.5-10}$ - Particulate matter with diameter between 2.5 and 10 microns; PM_{10} - Particulate matter with diameter < 10 microns; IQR - International Classification of Diseases, 9th revision

p-value: p-value of the interaction between PM_{10} and the Saharan dust indicator.

$PM_{2.5-10}$ data from NIH monitoring station; PM_{10} data from regional EPA monitoring stations.

Results from multivariate Poisson regression models adjusting for time trend, seasonality, day of the week, summer population decrease, holidays, influenza epidemics, high apparent temperatures (penalized spline, lag 0-1), low air temperatures (penalized spline, lag 1-6), barometric pressure (penalized spline, lag 0).

Supplemental Material, Figure 1.

(a)

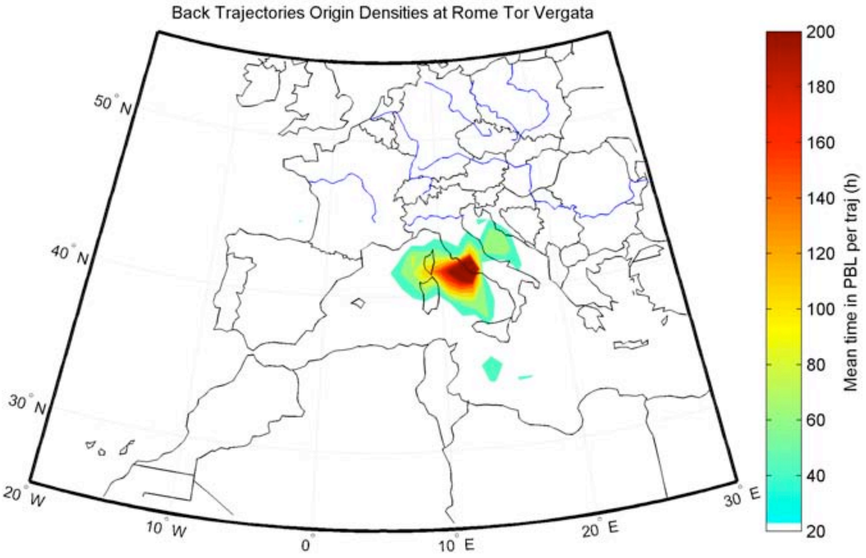

(b)

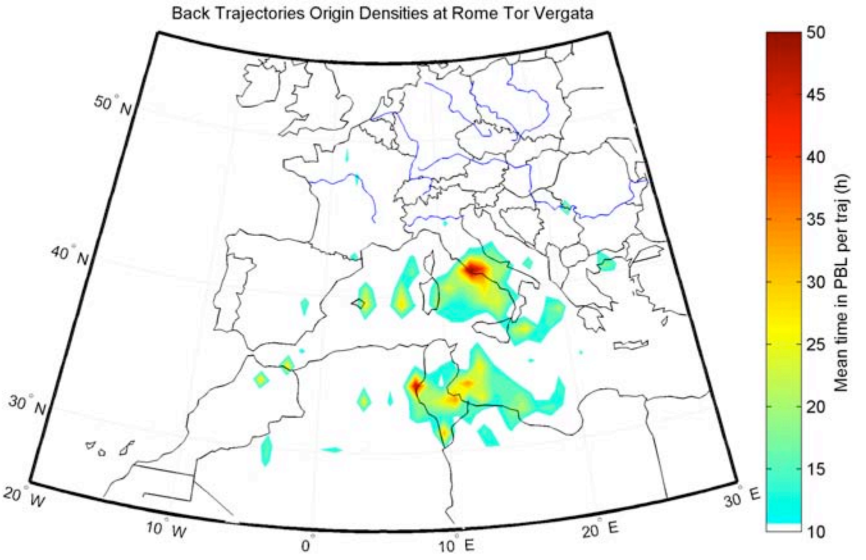

Supplemental Material, Figure 1. Contour plots of the total time (hours) spent in the Planetary Boundary Layer (PBL) by daily backtrajectories ending in Rome in the period 2001-2004, in non-dust (a) and dust (b) conditions.
